# Surface Modification of Calcined Kaolinite for Enhanced Solvent Dispersion and Mechanical Properties in Polybutylene Adipate/Terephthalate Composites

**DOI:** 10.3390/molecules29163897

**Published:** 2024-08-17

**Authors:** Yongbing Yuan, Xinyu Tang, Honghong Sun, Junkang Shi, Congshan Zhou, Derek O Northwood, Kristian E Waters, Hao Ma

**Affiliations:** 1 Department of Chemistry and Chemical Engineering, Hunan Institute of Science and Technology, Yueyang 414006, China; 2 BGRIMM Technology Group, Metallurgical Research and Design Institute, Beijing 100081, China; 3 Department of Mechanical, Automotive and Materials Engineering, University of Windsor, 401 Sunset Avenue, Windsor, ON N9B3P4, Canada; 4 Department of Mining and Materials Engineering, McGill University, 3610 University, Montreal, QC H3A 0C5, Canada

**Keywords:** calcined kaolinite, surface modification, 3-aminopropylriethoxysilane, phenyl glycidyl ether, PBAT, mechanical properties

## Abstract

In order to regulate the surface properties of calcined kaolinite for the purpose of achieving uniform distribution within various polar dispersion media, 3-aminopropyltriethoxysilane and phenyl glycidyl ether were employed to chemically modify calcined kaolinite. The grafting rate, surface properties, and dispersion properties of calcined kaolinite particles in different polar organic media were changed by varying the dosage of the modifiers. FT-IR analysis confirmed successful surface modification, while thermogravimetric analysis indicated a maximum graft coverage of 18.44 μmol/m^2^ for the modified particles. Contact angle measurements and particle size distribution analyses demonstrated the effective adjustment of surface characteristics by the modifiers. Specifically, at a mass ratio of 1.0 of modifier to kaolinite particles, the modified particles exhibited a contact angle of around 125°, achieving uniform dispersion in different polarity media. Particle size distribution ranged from 1600 nm to 2100 nm in cyclohexane and petroleum ether, and from 900 nm to 1200 nm in dioxane, ethyl acetate, and DMF, showcasing a significant improvement in dispersion performance compared to unmodified particles. Concurrently, to improve the mechanical properties of PBAT, modified particles were incorporated into the PBAT matrix, and the effect of modified particle addition on the tensile strength and fracture tensile rate of the composites was investigated. The optimal amount of modified particles is 6 wt.%~8 wt.%. This article aims at synthesizing modifier molecules containing different hydrophilic and hydrophobic groups to chemically graft onto the surface of calcined kaolinite. The hydrophilic and hydrophobic groups on the modified particles can adapt to dispersed systems of different polarities and achieve good distribution within them. The modified particles are added to PBAT to achieve good compatibility and enhance the mechanical properties of the composite material.

## 1. Introduction

In recent years, organic–inorganic composites have attracted the attention of many researchers because they tend to show unusual advantages in terms of mechanical strength and chemical and thermal stability compared to conventional materials [1,2,3,4,5,6,7,8]. Common inorganic particles used in the preparation of organic–inorganic composites are silica [4], titanium dioxide [6], zinc oxide [7,8], and clay minerals including montmorillonite [9,10,11] and kaolinite [12,13,14,15]. Clay minerals have attracted increasing attention since their first application in nylon in the 1980s due to their wide availability and low prices [16].

However, clay particles cannot be used directly in organic–inorganic composites due to their strong hydrophilicity on the surface. Thus, they need to be properly modified. Currently, research on the modification of clay particles mainly focuses on montmorillonite. The addition of modified montmorillonite particles to polymer molecules can significantly improve the fatigue resistance, flame retardancy, and dimensional stability of the materials [17,18,19,20]. Comparatively, there are fewer studies on the modification of kaolinite particles [21,22].

The challenge in kaolinite modification lies in the difficulty for larger modifier molecules to interact chemically with the aluminum hydroxyl groups in the interlayers due to strong van der Waals forces and hydrogen bonding between kaolinite layers [23,24]. To address this, small polar molecules are used as intercalators to expand interlayer spacing, easing the entry of modifier molecules [25]. Despite taking a prolonged time at ambient temperatures or requiring high temperatures, this method can be circumvented by using calcined kaolinite, which eliminates the need for intercalation and allows for direct chemical grafting with modifiers [26].

Recent studies often focus on converting kaolinite particle surfaces to hydrophobic surfaces to enhance compatibility with polymer matrices. However, a more targeted modification is essential to cater to various solvents used in composite production. This study aims to develop a straightforward and efficient method to adjust the surface properties of calcined kaolinite particles for uniform dispersion in different polar media [27]. Polybutylene adipate/terephthalate (PBAT) is a biodegradable plastic with good plasticity, flexibility, and heat and cold resistance [28,29,30,31,32,33]. However, since the mechanical properties of PBAT are poor, it is necessary to find appropriate methods to improve its mechanical properties. The preparation of composites by adding reinforcement to the matrix material is an effective method to improve the mechanical properties [34]. The selection of suitable reinforcing agents to prepare composites with PBAT, which retains the biodegradability of PBAT and effectively improves the mechanical properties of PBAT, is a potentially feasible way to produce mulch film, biodegradable plastic bags, disposable tableware, and medical gauze [35,36,37,38,39].

In this study, 3-aminopropyltriethoxysilane (APTES) and phenyl glycidyl ether (PGE) were used to chemically modify the calcined kaolinite particles. The surface properties of calcined kaolinite particles were effectively regulated by the alkyl, hydroxyl, phenyl, and amino groups preserved on the molecules of the modifiers, while the dispersion properties of the modified particles in different polar dispersing media were explored through contact angle tests, particle size analysis, and SEM analysis, and the mechanical properties of the modified particles/PBAT composites were analyzed through ductility and tensile strength tests.

## 2. Results and Discussion

### 2.1. FT-IR Spectrograms

**Figure 1:**
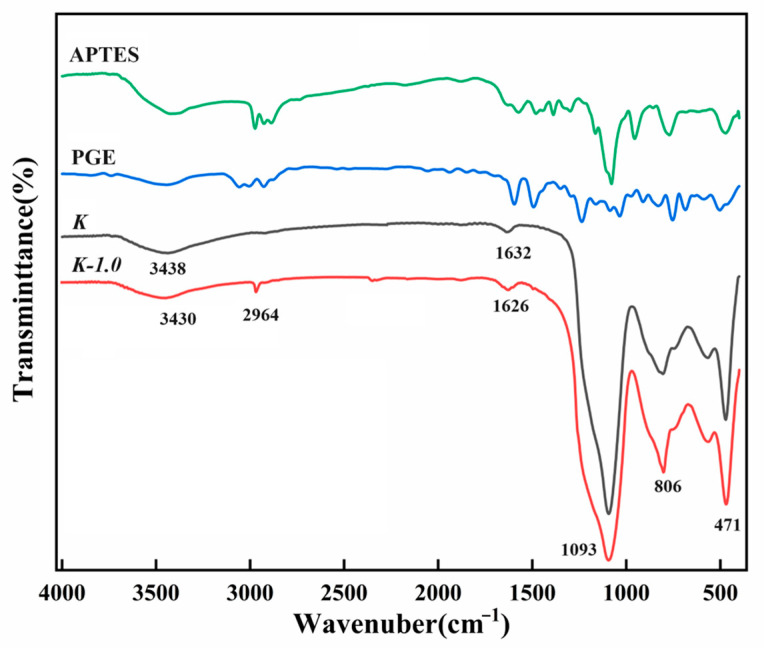
Infrared spectra of APTES, PGE, K, and K-1.0.

The FTIR spectra of APTES, PGE, K, and K-1.0 are displayed in Figure 1. In the spectrum of the K sample, two absorption vibrational peaks at around 1093 cm^−1^ and 471 cm^−1^ are identified as Si-O symmetric stretching and Si-O-Si bending vibrational peaks, respectively. The peak at 806 cm^−1^ corresponds to Al-O vibration in kaolinite [40,41]. The -OH stretching peak is observed at 3438 cm^−1^, while the adsorbed water O-H bending peak appears at 1632 cm^−1^. In comparison, the K-1.0 sample exhibits a new peak at 2964 cm^−1^ attributed to -CH_2_ stretching and a weak symmetric -CH_3_ bending peak near 1497 cm^−1^. The emergence of these peaks indicates the chemical grafting of the modifier resulting from the reaction between APTES and PGE on the surface of calcined kaolinite particles. Furthermore, in comparison to the K sample, the -OH peak of K-1.0 migrated to a lower band (~3430 cm^−1^), and the deformation vibration peak of O-H also shifted towards lower wave numbers (~1626 cm^−1^). This migration further confirmed the presence of -NH- groups on the K-1.0 particles [42].

**Figure 2:**
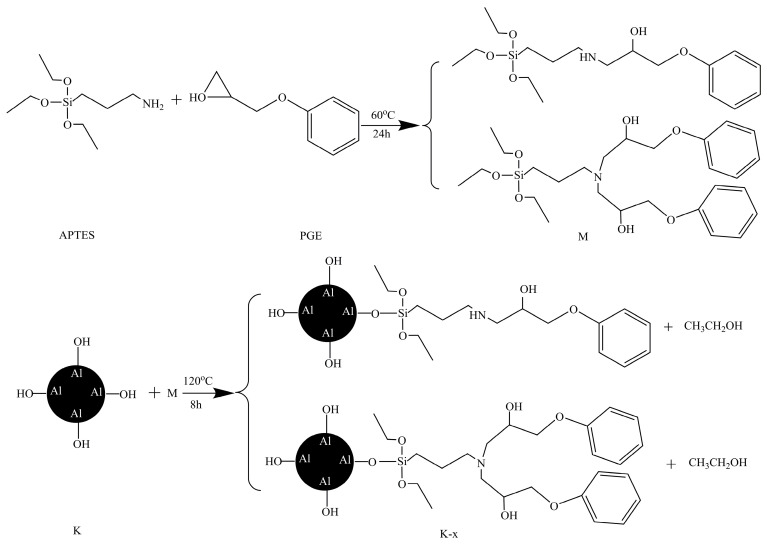
Reaction mechanism diagram.

Since PGE cannot directly react with calcined kaolinite, it can be inferred that APTES first reacts with PGE to generate the modifier M. When M reacts with calcined kaolinite, RO-Si in the modifier molecule first hydrolyzes to form silanol groups (Si–OH), and subsequent condensation reactions occur between silanol groups (Si–OH) and Al-OH located on the surface of calcined kaolinite to form covalent bonds (Al-O-Si) [14,26,43], as shown in Figure 2. It should be noted during this process, the C-O bond breaks to form an O-H bond. The bond energy associated with the C-O bond is approximately 360 kJ/mol, while the bond energy for the O-H bond is around 430 kJ/mol [44]. After chemical modification, the surface of calcined kaolinite particles is covered with benzene ring, hydroxyl, and alkyl groups, and thus its surface properties will be changed.

### 2.2. Thermogravimetric Analysis

**Figure 3:**
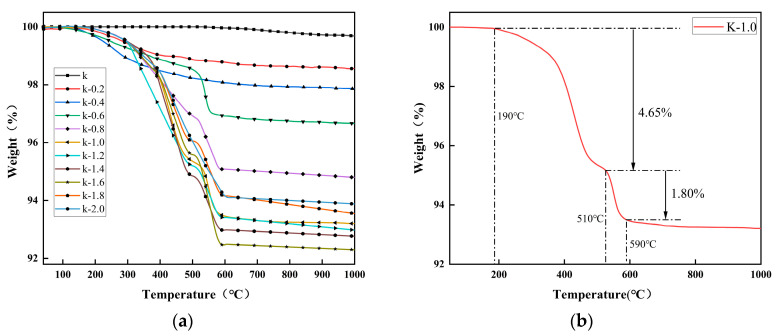
Thermogravimetric analysis of (**a**) unmodified and modified calcined kaolinite (**b**) the K-1.0 samples.

Figure 3 displays the thermogravimetric analysis results of unmodified and modified calcined kaolinite particles. In Figure 3a, the weight losses of modified calcined kaolinite (1.44–7.70%) were higher than those of blank calcined kaolinite (0.35%), suggesting successful chemical grafting on the surface of the kaolinite particles with a significant number of modifier molecules attached. The degree of thermogravimetric loss variation was more pronounced for x values between 0.2 and 1.0 compared to x values between 1.0 and 2.0. This could be attributed to a fixed number of reactive sites when the mass of calcined kaolinite remains constant. When the amount of modifier equals the mass of calcined kaolinite, most reactive sites on the particle surface are chemically grafted, limiting further grafting even with increased modifier amounts. Figure 3b illustrates the thermogravimetric analysis of K-1.0 samples. A weight loss of 4.65% between 190 and 510 °C may result from carbon chain breakage in the modified molecules grafted onto the particle surface. Additionally, the weight loss observed at 510~590 °C likely corresponds to the benzene ring group decomposition within the modifier molecules, accounting for approximately 1.80% of the total weight loss. Thermogravimetric data can also quantify the graft coverage of the modifier on the surface of kaolinite particles [45,46], which can be estimated by Equations (1)–(4).

(1) $$N_{t}=\frac{2\Delta m_{K}}{M_{w}}$$

(2) $$N_{r}+N_{u}=N_{t}$$

(3) $$N_{r}\times M+\frac{N_{u}}{2}\times M_{w}=\Delta m_{K-x}$$

(4) $$S_{C}=10^{6}\frac{N_{r}}{SSA}$$

**Figure 4:**
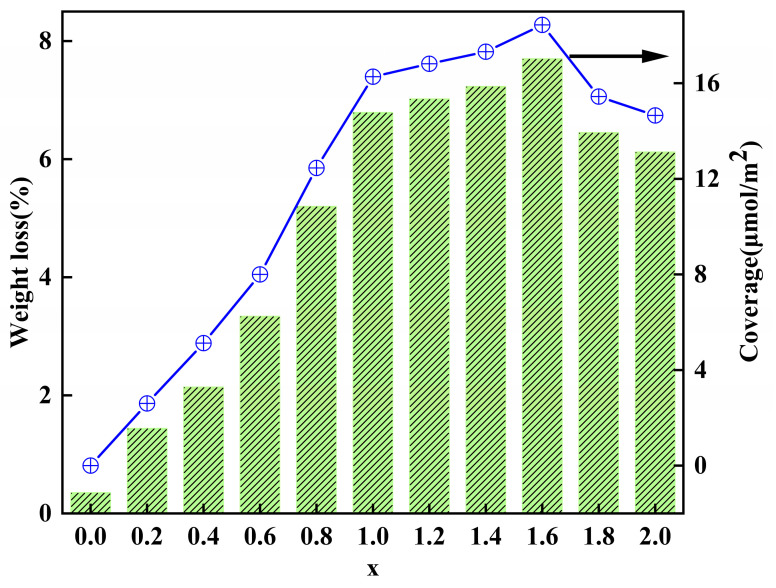
Thermogravimetric weight loss and surface graft coverage of unmodified and modified kaolinite.

In Equations (1)–(4), N_t_, N_r_, and N_u_ represent the amount of substance of all, reacted and unreacted, hydroxyl groups on the calcined kaolinite particles (mol/g), respectively; Δm_K_ and Δm_K-x_ denote the total mass loss of K and K-x (g/g); M_w_ is the molecular weight of water, 18 g/mol; M denotes the average molecular mass of the modifier (342 g/mol); S_c_ denotes the graft coverage (μmol/m^2^); and SSA denotes the specific surface area of K, 12.5 m^2^/g. The analytical results are shown in Figure 4.

In Figure 4, it is evident that as the modifier dosage increased, the grafting coverage and thermal weight loss rate also increased simultaneously. Particularly for x values between 0.2 and 1.0, these two parameters showed rapid escalation, while for x values between 1.2 and 1.6, both the grafting coverage and thermal weight loss rate exhibited slower growth and peaked at 18.44 μmol/m^2^ and 7.7%, respectively. This indicates that the grafting process neared completion and the reaction sites on the calcined kaolinite particles were nearly exhausted. Upon further increase in x, the grafting coverage and thermogravimetric loss rate declined. This decline can be attributed to the accumulation of previously grafted modifier groups on the kaolinite particle surface, creating significant spatial site resistance. This resistance hinders the approach of additional modifier molecules to the remaining reaction sites, impeding the grafting reaction and leading to a reduction in the grafting rate instead.

### 2.3. Contact Angle

**Figure 5:**
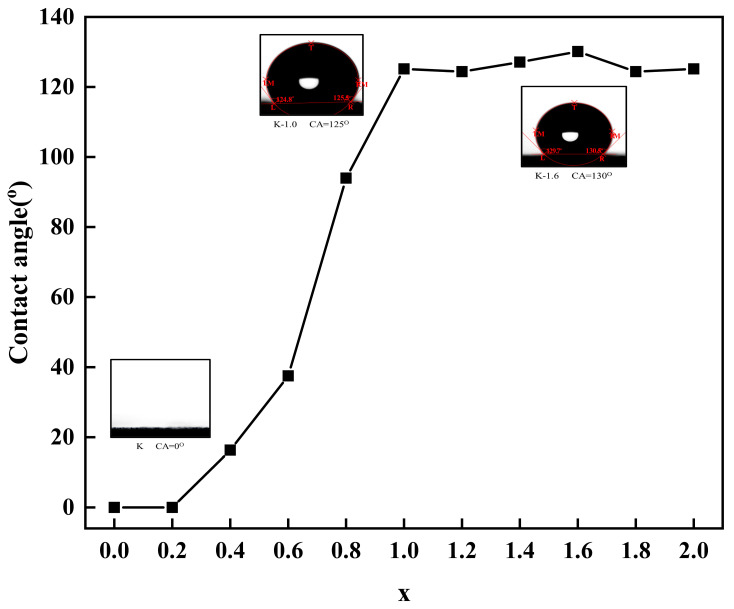
Contact angle of unmodified and modified kaolinite powder.

The contact angles of unmodified and calcined kaolinite powders were measured to determine the changes in the surface properties of kaolinite, and the results are shown in Figure 5.

In Figure 5, it is illustrated that the contact angle of the modified particles gradually increases as the amount of modifier M increases. Within the range of x = 0.2~1.0, the contact angle shows a rapid incline, reaching approximately 125° for the K-1.0 sample. For x values exceeding 1.0, the contact angle stabilizes. This observation suggests that the modifier coating on the calcined kaolinite particle surface alters its surface properties. As the modifier amount increases, the particle surface’s hydrophilicity decreases while its hydrophobicity increases. However, there is a saturation point for these changes in hydrophilicity and hydrophobicity, as evidenced by the thermogravimetric analysis in Figure 3. When a majority of hydroxyl groups on the calcined kaolinite surface are grafted, any excess modifier cannot further graft onto the particle surface, leading to a constant contact angle.

### 2.4. Dispersion Performance

**Figure 6:**
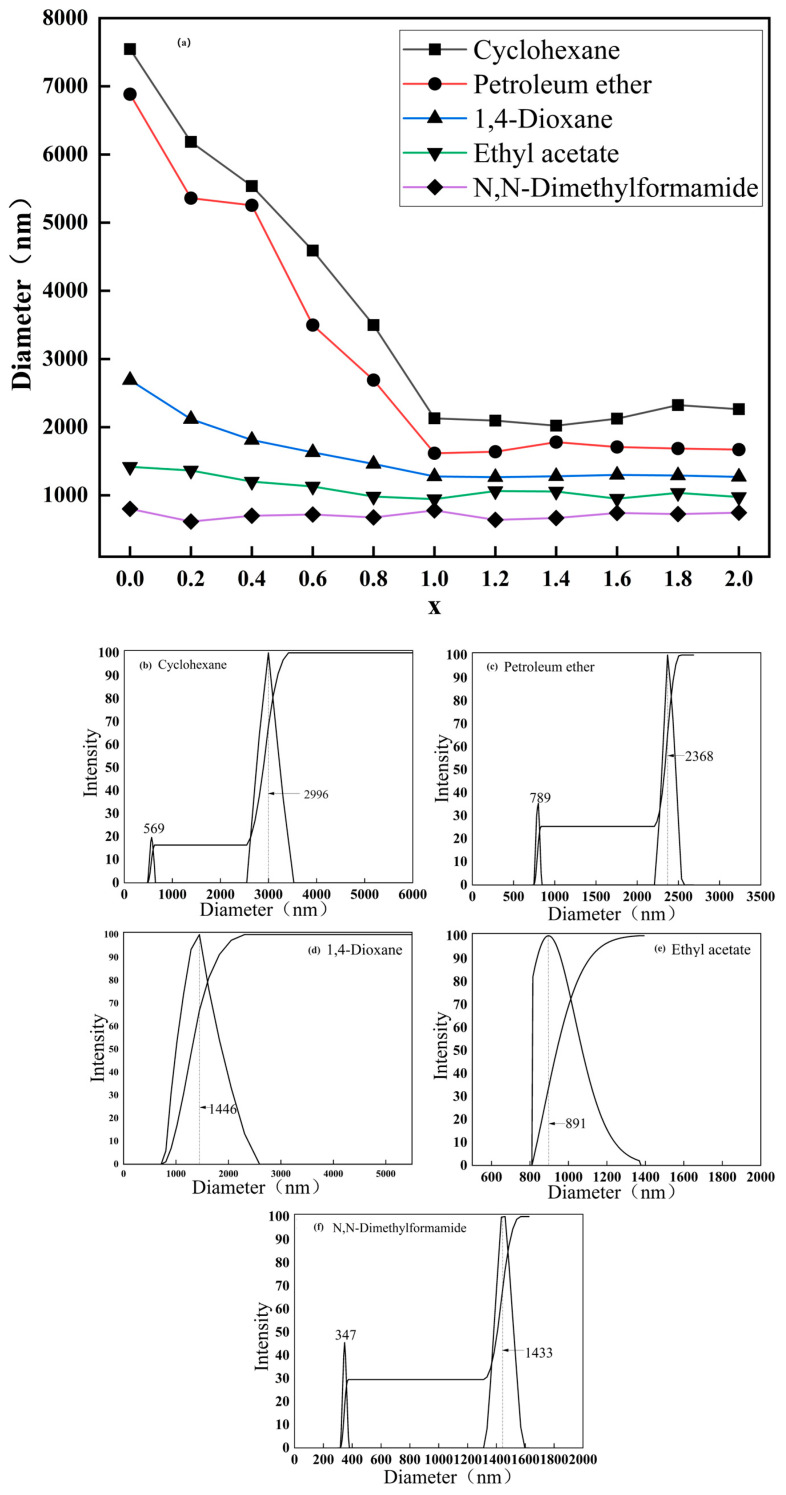
Average particle size of K and K-x particles in (**a**) different polar dispersion media; the particle size distribution of K-1.0 in (**b**) cyclohexane; (**c**) petroleum ether; (**d**) 1,4-dioxane; (**e**) ethyl acetate; and (**f**) N,N-dimethylformamide.

**Figure 7:**
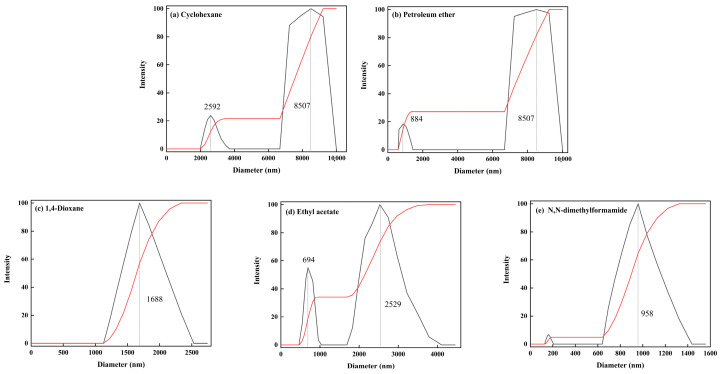
The particle size distribution of the K sample in different dispersion medias: (**a**) cyclohexane; (**b**) petroleum ether; (**c**) 1,4-dioxane; (**d**) ethyl acetate; (**e**) N,N-dimethylformamide.

K and K-x were dispersed in media with different polarities using a high-speed homogenizer, and then their average particle size and size distribution were determined using a particle size analyzer to judge the dispersion performance of the particles in the media. The results are shown in Figure 6 and Figure 7.

Figure 6a compares the average particle sizes of unmodified (x = 0) and modified (x = 0.2, 0.4, 0.6, 0.8, 1.0, 1.2, 1.4, 1.6, 1.8, 2.0) calcined kaolinite particles in various dispersion systems: cyclohexane, petroleum ether, 1,4-dioxane, ethyl acetate, and DMF. The average sizes of unmodified particles in these systems were 7500 nm, 6800 nm, 2600 nm, 1400 nm, and 900 nm, respectively. Unmodified particles tend to agglomerate in nonpolar systems due to their strong hydrophilicity, resulting in larger sizes compared to polar solvents. In contrast, modified particles generally exhibited smaller sizes in polar solvents, with the most significant reduction observed within the x = 0.2~1.0 range, plateauing beyond x > 1.0. Notably, the K-1.0 sample consistently displayed the smallest average size across most media, notably shrinking from 7500 nm and 6800 nm to 2100 nm and 1600 nm in cyclohexane and petroleum ether. Moreover, the chemical modification of calcined particles led to improved dispersion in various polar systems, particularly enhancing performance in nonpolar environments. This enhancement is attributed to the diverse surface functional groups introduced during modification, promoting uniform distribution in different media.

Figure 6b–f depicts the particle size distribution of K-1.0 particles in different polar solvents. The particles showed more uniform distribution in 1,4-dioxane and ethyl acetate, with median sizes of 1446 nm and 891 nm, respectively. While DMF exhibited a smaller average size, distinct peaks at 347 nm and 1433 nm were evident. Similar peaks were observed in cyclohexane and petroleum ether. Given K-1.0’s excellent performance in all dispersion media, it was selected as an additive for preparing the composite material P-y with PBAT. Figure 7 shows the particle size distribution of K sample in different dispersion medias.

The distribution analysis reveals that K demonstrates optimal dispersion in highly polar organic solvents, characterized by small particle sizes and a concentrated distribution. In contrast, in weakly polar solvents like cyclohexane and petroleum ether, K exhibits poor distribution with varying particle sizes and significant agglomeration, evident by large agglomerates reaching up to 10,000 nm. This uneven distribution pattern forms a bimodal distribution in these solvents. A comparison between K-1.0 and K samples in the same solvent shows that K-1.0 significantly improves distribution in weakly polar solvents, with peak sizes decreasing from 8507 nm to around 3000 nm and 2400 nm in cyclohexane and petroleum ether. Likewise, in dioxane and ethyl acetate, peak sizes decrease from 1688 nm and 2529 nm to 1446 nm and 891 nm, respectively. The appropriate chemical modification of calcined kaolinite’s surface enables it to adapt to various polar solvents, enhancing its dispersibility.

### 2.5. Scanning Electron Microscopy of Unmodified and Modified Kaolinite

**Figure 8:**
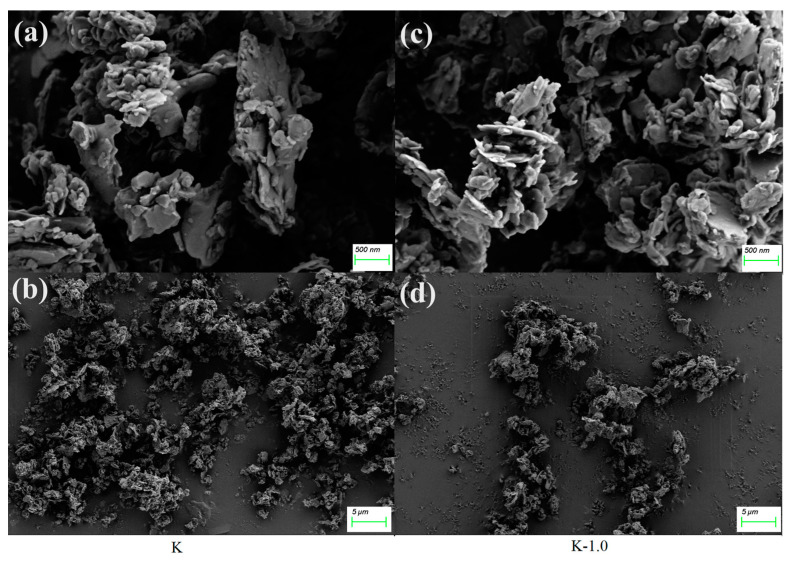
Scanning electron microscopy images of K (**a**,**b**) and K-1.0 (**c**,**d**).

The SEM images in Figure 8 depict the comparison between unmodified K and modified K-1.0. Figure 8a,c show the direct analysis of the untreated and modified particles, respectively, while Figure 8b,d display dispersions formed in cyclohexane for both samples after ultrasonication. From Figure 8a,c, it can be seen that compared to the layered structure of ordinary kaolinite mentioned in the previous literature [47,48], calcined kaolinite is in a disordered state, indicating that after high-temperature calcination, the majority of the layered structure has disintegrated and no longer exists. Figure 8b illustrates that most untreated calcined kaolinite particles tend to aggregate, whereas in Figure 8d, modified K-1.0 particles exhibit improved dispersion in cyclohexane with reduced agglomeration. This trend aligns with the particle size analysis in Section 2.4, suggesting that appropriate modification enhances the hydrophilic interaction between calcined kaolinite particles and non-polar dispersing media, facilitating uniform dispersion.

### 2.6. Mechanical Properties of Composites

**Figure 9:**
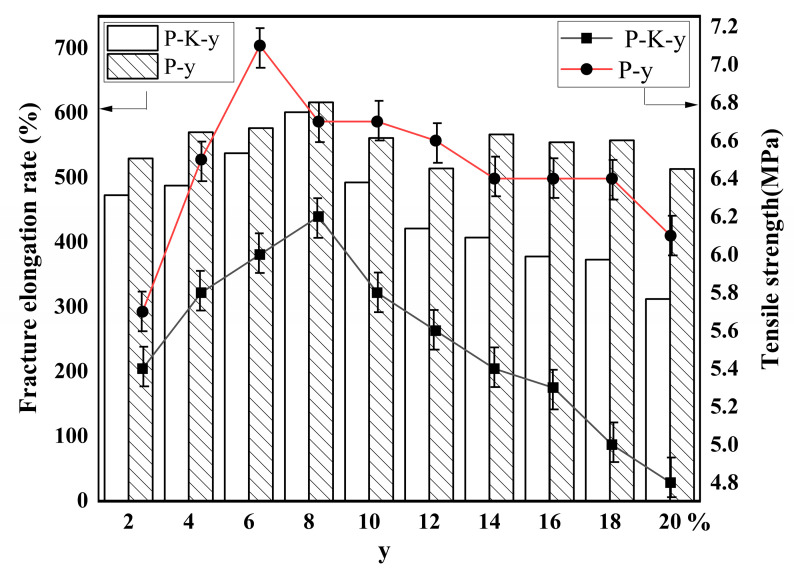
Elongation to fracture and tensile strength of P-y and P-K-y composite materials.

The mechanical properties of P-K-y and P-y composites are shown in Figure 9.

In Figure 9, it is observed that the tensile strength of P-y exceeds that of P-K-y at the same additive level, peaking at around 7 MPa with a 6% modified particle content. As the amount of modified particles surpasses 6%, tensile strength diminishes. Similarly, the fracture tensile rate and ductility of P-y outperform P-K-y, with the maximum ductility achieved at an 8% additive content. Ductility decreases beyond this threshold. The addition of modified particles to PBAT enhances the mechanical properties by ensuring homogeneous dispersion within the matrix material, unlike unmodified particles, which tend to agglomerate, limiting their impact on mechanical properties. The quantity of modified particles should be carefully controlled; exceeding 6% leads to a gradual reduction in tensile strength, as excessive amounts weaken molecular chain connections in PBAT, causing whitening and brittleness. Compared with common PLA/PBAT blends [49] and PHBV/PBAT blends [50], the modified calcined kaolinite particles have excellent compatibility with PBAT at a lower dosage, thereby significantly improving the mechanical properties, including tensile strength and fracture tensile rate. An optimal mechanical and cost-effective balance is achieved at a 6–8% modified particle content for improved composite performance.

## 3. Experiments and Analysis

### 3.1. Materials

The elemental analysis of calcined kaolinite (referred to as K, 6000 mesh, specific surface area of 12.5 m^2^/g) is given in Table 1:

The calcined kaolinite used in this work was purchased from Shanghai Aladdin Biochemical Technology Co., Ltd. (Shanghai, China), and the calcination temperature was 1000 °C. The sample was not been subjected to any additional laboratory processing before the experiment. Analytically pure grades of N, N-dimethylformamide (DMF), dichloromethane (DCM), anhydrous ethanol, cyclohexane, ethyl acetate, dioxane, petroleum ether, 3-aminopropyltriethoxysilane (APTES, 99%), and phenyl glycidyl ether (PGE, 99%) were purchased from Aladdin Reagent Shanghai Co. (Shanghai, China). Polybutylene adipate/terephthalate (PBAT) was purchased from Shanghai McLean Biochemical Technology Co. (Shanghai, China).

### 3.2. Preparation of Modified Calcined Kaolinite/PBAT Composites

(1) The modifier was prepared by mixing 100 mL of DMF, 70.91 mL (0.30 mol) APTES, and 82.07 mL (0.60 mol) PGE in a flask, followed by stirring the mixture at 60 °C for 24 h.

(2) Modified calcined kaolinite was obtained by drying K at 100 °C for 4 h, then adding 4.0000 g of K to 100 g of DMF along with 0.8000 g of a modifier M (K:M mass ratio of 1:0.2). The mixture was stirred at 120 °C for 8 h, followed by centrifugal washing with anhydrous ethanol five times. The resulting product, named K-0.2, was dried at 80 °C for 24 h. Different amounts of M were used to produce products denoted as K-x, where x = 0.4, 0.6, 0.8, 1.0, 1.2, 1.4, 1.6, 1.8, and 2.0.

(3) Composite preparation involved placing 10.00 g of PBAT in a beaker, adding 100 mL of DCM, and stirring for 30 min for complete dissolution. Next, 0.20 g of modified particles was dispersed in the DCM using a high-speed homogenizer. The mixture was then poured into a glass Petri dish, sealed with plastic wrap, and air-dried for 24 h with small holes in the wrap. Subsequently, it was dried in a 60 °C oven for 1 h to produce the composite material, labeled as P-2%. Varying amounts of modified particles were used to prepare composites denoted as P-y, where y = 4%, 6%, 8%, 10%, 12%, 14%, 16%, 18%, and 20%. Composites using unmodified particles were denoted as P-K-y, obtained through similar processes.

### 3.3. Analytical Characterization

The FT-IR spectra of the samples were obtained using a Nicolet Avatar 370 FT-IR spectrometer (Thermo Fisher, San Diego, CA, USA) with a wave number range of 400~4000 cm^−1^ and a resolution of 2 cm^−1^. Thermogravimetric analysis was carried out using a NETZSCH STA 449F3 thermogravimetric analyzer (Netzsch, Hanau, Germany), with a flow rate of nitrogen of 40 mL/min and a heating rate of 10 °C/min, and a temperature range of 40~1000 °C. The contact angle of the samples was tested using a CA100 contact angle analyzer. The distribution of the samples in various polar and non-polar media was determined using a NanoBrook 90 plus PALS multi-angle particle size analyzer (Brookhaven Instrument, Holtsville, NY, USA), and the samples were completely dispersed in a dispersing reagent using a high-speed homogenizer running at 25,000 r/min for 1 min before each determination. The surface morphology of the samples was observed using a Zeiss Sigma 300 field emission scanning electron microscope (Zeiss, Oberkochen, Germany). The tensile strength of the composites was tested using an FR-103C universal material tensile tester, and the fracture tensile rate was measured with loading rate of 10.0 mm min^−1^.

## 4. Conclusions

In this study, the surface of calcined kaolinite particles was chemically modified using APTES and PGE, and the surface properties of modified kaolinite particles were optimized by changing the ratio between the modifier and kaolinite particles during the grafting process. A uniform distribution of particles could therefore be achieved in both polar and nonpolar dispersion systems. Organic groups such as alkyl, phenyl, hydroxyl, and sec-amino groups coated on the surface of the modified particles can improve the phophilicity between the modified particles and different dispersing media. This greatly reduces the tendency for agglomeration of the calcined kaolinite particles in non-polar solvents, so as to enable the modified particles to achieve homogeneous dispersion in cyclohexane, petroleum ether, 1,4-dioxane, ethyl acetate, and DMF. Compared with the unmodified kaolinite particles, the physical properties of the composites prepared from the modified particles K-1.0 and PBAT were effectively improved. The tensile strength of the composites was maximized when the addition amount was 6%, and the ductility of the composites was maximized when the amount added was 8%.

## Figures and Tables

**Table 1:** Chemical composition of the calcined kaolinite.

Element	Fe_2_O_3_	TiO_2_	CaO	K_2_O	P_2_O_5_	MgO	Na_2_O	Al_2_O_3_	SiO_2_
Con. (%)	0.419	0.678	0.177	0.263	0.382	0.206	0.424	49.48	47.89

## Data Availability

The original contributions presented in the study are included in the article, further inquiries can be directed to the corresponding author.
